# A Novel Pyroptosis-Related Gene Signature for Predicting Prognosis in Kidney Renal Papillary Cell Carcinoma

**DOI:** 10.3389/fgene.2022.851384

**Published:** 2022-03-23

**Authors:** Jian Hu, Yajun Chen, Liang Gao, Chengguo Ge, Xiaodu Xie, Pan Lei, Yuanfeng Zhang, Peihe Liang

**Affiliations:** ^1^ Department of Urology, The Second Affiliated Hospital of Chongqing Medical University, Chongqing, China; ^2^ Department of Hepatobiliary Surgery, The Second Affiliated Hospital of Chongqing Medical University, Chongqing, China

**Keywords:** pyroptosis, kidney renal papillary cell carcinoma, signature, prognosis, gene

## Abstract

Pyroptosis is defined as an inflammatory form of programmed cell death. Increasing studies have demonstrated that pyroptosis is closely related to tumor development and antitumor process. However, the role of pyroptosis in kidney renal papillary cell carcinoma (KIRP) remains obscure. In this study, we analyzed the expression of 52 pyroptosis-related genes (PRGs) in KIRP, of which 20 differentially expressed PRGs were identified between tumor and normal tissues. Consensus clustering analysis based on these PRGs was used to divided patients into two clusters, from which a significant difference in survival was found (*p* = 0.0041). The prognostic risk model based on six PRGs (*CASP8*, *CASP9*, *CHMP2A*, *GPX4*, *IL6*, and *IRF1*) was built using univariate Cox regression and LASSO–Cox regression analysis, with good performance in predicting one-, three-, and five-year overall survival. Kaplan–Meier survival analysis showed that the high-risk group had a poor survival outcome (*p* < 0.001) and risk score was an independent prognostic factor (HR: 2.655, 95% CI 1.192–5.911, *p* = 0.016). Immune profiling revealed differences in immune cell infiltration between the two groups, and the infiltration of M2 macrophages was significantly upregulated in the tumor immune microenvironment, implying that tumor immunity participated in the KIRP progression. Finally, we identified two hub genes in tumor tissues (*IL6* and *CASP9*), which were validated *in vitro.* In conclusion, we conducted a comprehensive analysis of PRGs in KIRP and tried to provide a pyroptosis-related signature for predicting the prognosis.

## Introduction

Renal cell carcinoma (RCC) is one of the most common tumors in the genitourinary system, accounting for 3.7% of all malignancies globally (Sung et al., 2021). Kidney renal papillary cell carcinoma (KIRP) refers to a subtype of RCC, with a relatively lower invasiveness and better prognosis than other types of RCC. However, approximately 25–35% of RCC patients had distant metastasis at the time of initial diagnosis, and the five-year survival rate of metastatic RCC was found to be only about 12% (Brozovich et al., 2021; Roberto et al., 2021). Accordingly, a novel risk model should be developed to identify potential high-risk KIRP patients, which may be conductive to clinical decision-making or exploring novel therapeutic biomarkers.

Pyroptosis has been reported as an inflammatory type of programmed cell death mediated by gasdermin proteins (Xia et al., 2019). The members of gasdermin families consist of GSDMA, GSDMB, GSDMC, GSDMD, GSDME (or DFNA5), and PJVK (or DFNB59) (Broz et al., 2020). Pyroptosis is characterized by pore formation in the plasma membrane, which can lead to the formation of inflammasomes and the release of pro-inflammatory factors, thus resulting in cell death. Pyroptosis was firstly discovered in the inflammatory response to infection (Zychlinsky et al., 1992). According to further research studies, more functions relating to pyroptosis in neurological, infectious, autoimmune, cardiovascular, and oncologic disorders have been found (Yu et al., 2021). Over the past few years, increasing studies have confirmed that pyroptosis might play a double-edged role since it could both promote and inhibit tumor cells. On the one hand, the activated pyroptosis can result in the release of inflammatory mediators, such as IL-1 and IL-8, which can form an inflammatory environment and facilitate the occurrence of cancer (Chavez-Dominguez et al., 2021). On the other hand, inducing pyroptosis of tumor cells showed a great potential in inhibiting tumor proliferation, migration, and invasion (Derangere et al., 2014; Wang et al., 2017). For example, iron-activated reactive oxygen species (ROS) could promote melanoma cell pyroptosis *via* a Tom20–Bax–caspase–GSDME pathway (Zhou et al., 2018). However, the effect of pyroptosis on the development and prognosis of KIRP remains unknown.

In this study, we performed a comprehensive analysis for the expression level of pyroptosis-related genes (PRGs) in KIRP and constructed a signature to predict the survival outcomes of KIRP patients. Subsequently, functional enrichment analysis and its interactions with cancer immunity of the signature were further explored. Furthermore, hub genes of the signature were validated.

## Materials and Methods

### Dataset Acquisition

The normalized RNA sequencing (RNA-seq) expression data as transcripts per million (TPM) and corresponding clinical information of 321 KIRP samples were acquired from TCGA database (https://xenabrowser.net/datapages/, until December 01, 2021). After screening, the 72 samples were rejected based on the merged sample quality annotations (https://gdc.cancer.gov/about-data/publications/pancanatlas). Additionally, 28 normal kidney samples were collected from the Genotype-Tissue Expression (GTEx) database (https://xenabrowser.net/datapages/, until December 01, 2021). All RNA-seq data were log_2_-transformed for further analysis.

### Identification of Differentially Expressed PRGs

As shown in Supplementary Table S1, the 52 PRGs were retrieved from GSEA (http://www.gsea-msigdb.org/gsea/index.jsp) and previous research (Supplementary Table S1) (Qi et al., 2021). The “limma” R package was utilized to determine differentially expressed genes (DEGs) between 28 normal kidney samples and 249 KIRP samples, with |Log_2_FC|>1 and *p* < 0.05. The differentially expressed PRGs were selected through the “VennDiagram” package, and their protein–protein interaction (PPI) network was acquired from the STRING database (https://www.string-db.org/, version 11.5).

### Consensus Clustering Analysis

To investigate the biological characteristics of differentially expressed PRGs in KIRP patients, we classified the patients into different subtypes using the “ConsensusClusterPlus” R package with a resampling rate of 80% and 500 iterations. The differential clinical information and expression of different subtypes were shown in the heat-map. The survival differences among clusters were visualized with the Kaplan–Meier curve using the “survival” R package.

### Establishment of a Pyroptosis-Based Prognostic Model

Univariate Cox proportional hazards regression analysis determined the prognostic value of PRGs in KIRP patients, and genes with *p* < 0.2 were selected for subsequent analysis. The candidate PRGs were selected using 10-fold cross-validation of the least absolute shrinkage and selection operator (LASSO)-penalized Cox regression analysis in the “glmnet” R package. Then, the prognostic model was built based on the six genes (*CASP8*, *CASP9*, *CHMP2A*, *GPX4*, *IL6*, and *IRF1*) and their coefficients, and the penalty parameter (λ) was decided by the minimum criteria. The risk score of each patient was calculated according to regression coefficients derived from the LASSO-Cox regression model multiplied with its gene expression level, as follows: Risk score = $\overset{6}{\underset{\text{i}}{\sum }}\text{Xi*Yi}$ (X: coefficients, Y: gene expression level). Next, 249 patients were separated into the low-risk group and the high-risk group based on the median risk score, and Kaplan–Meier survival analysis and a log-rank test were performed to compare the survival outcomes between two risk groups. The area under the curve (AUC) of the receiver-operating characteristic (ROC) curve based on the “survival,” “survminer,” and “time-ROC” R packages was used to evaluate the predictive performance of the prognostic model.

### Independent Prognostic Analysis of Risk Scores

To identify independent prognostic factors and validate the independent prognostic value of risk score, the risk score and clinical characteristics including age, gender, and T-stage, N-stage, M-stage, and tumor stage in TCGA dataset were analyzed *via* univariate and multivariate Cox regression models, respectively. These multivariate prognostic analysis results were calculated and then visualized by the “forestplot” R package.

### Functional Enrichment Analysis of DEGs and Evaluation of Tumor Immune Microenvironment

The DEGs between the low-risk group and the high-risk group were identified *via* the “limma” R package. |Log_2_FC|>1 and *p* < 0.05 were considered to be statistically significant. The Gene Ontology (GO) and Kyoto Encyclopedia of Genes and Genomes (KEGG) enrichment analyses of those DEGs were performed *via* the “clusterProfiler” R package. The “CIBERSORT” package was used to explore the landscape of 22 tumor-infiltrating immune cells and their connection with the signature.

### Ethics Statement and Tissue Sample Collection

A total of three pairs of tissues from KIRP patients and their paired normal tissues were collected from the Department of Pathology of The Second Affiliated Hospital of Chongqing Medical University, which was approved by the Human Research Ethics Committee.

### Immuno-Histochemical Staining

Paraffin sections were placed in a 60°C oven to melt the paraffin and soaked in xylene and ethanol at different concentrations to elute the paraffin. Then, the sections were incubated with 3% H_2_O_2_ at room temperature for 10 min to eliminate endogenous peroxidase activity. The sections were immersed in boiling EDTA repair solution for 10 min and allowed to cool naturally. Then, the sections were incubated with 5% BSA blocking solution at 37°C for 30 min. The sections were incubated with appropriately IL-6 primary antibody (1:50, Proteintech, 21865-1-AP) and CASP9 primary antibody (1:200, Abcam, ab202068) at 4°C overnight. The next day, the sections were washed three times with PBS for 10 min. The sections were incubated with secondary antibody at room temperature for 60 min. After washing three times with PBS for 10 min, the tissues were stained with DAB and hematoxylin. Then, the sections were sequentially immersed in 60, 75, 80, 95, and 100% ethanol for dehydration. Finally, the sections were sealed with neutral gum and observed with a light microscope.

### Statistical Analysis

The DEGs between the normal and KIRP tissues were analyzed with one-way analysis of variance. The Kaplan–Meier curve with a two-sided log-rank test was utilized to assess the survival difference. Cox regression models were applied to identify prognostic factors, with hazard ratios (HRs) and 95% confidence intervals (CIs). All statistical analyses were completed by R software (v4.1.0), and *p* < 0.05 was considered statistically significant.

## Results

### Identification of Differentially Expressed PRGs Between KIRP and Normal Tissues

**FIGURE 1:**
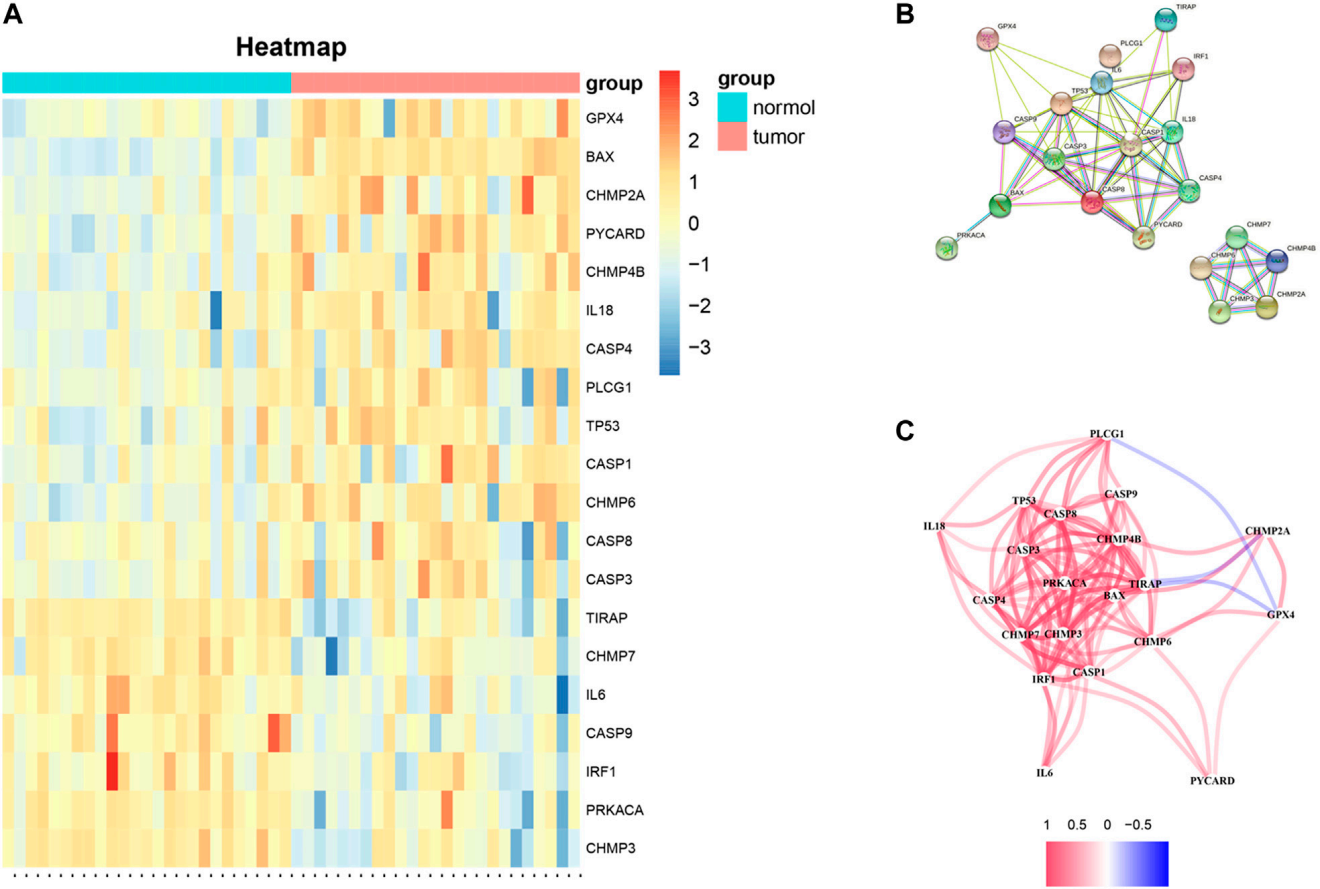
**(A)** Heat-map of differentially expressed PRGs between the tumor and normal tissues (red color: higher expression; blue color: lower expression, all *p* < 0.05). **(B)** PPI network of 20 differentially expressed PRGs obtained from the STRING database. **(C)** Correlation network of 20 differentially expressed PRGs (the correlation coefficients are presented by different colors: red line, positive correlation; blue line, negative correlation).

The mRNA expression of 52 PRGs from 249 tumor and 28 normal tissues was examined on the basis of TCGA data. 20 genes were considered differentially expressed PRGs with |Log2FC|>1 and *p* < 0.05. As shown in Figure 1A, 13 genes (*GRX4*, *BAX*, *CHMP2A*, *PYCARD*, *CHMP48*, *IL18*, *CASP4*, *PLCG1*, *TP53*, *CASP1*, *CHMP6*, *CASP8*, and *CASP3*) of the above genes were upregulated, and 7 genes (*TIRAP*, *CHMP7*, *IL6*, *IRF1*, *CASP9*, *PRKACA*, and *CHMP3*) were downregulated in the tumor group. To further explore the interactions of these 20 differentially expressed PRGs, PPI network analysis was conducted, and the results are shown in Figure 1B. And the correlation network of these genes is shown in Figure 1C.

### Consensus Clustering Analysis Based on Differentially Expressed PRGs

**FIGURE 2:**
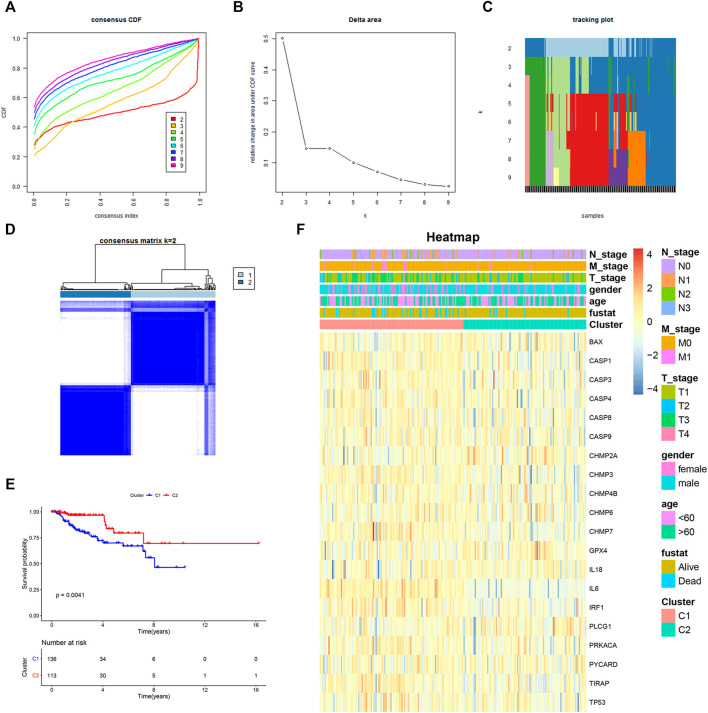
Two KIRP clusters obtained by consensus clustering analysis based on differentially expressed PRGs. **(A)** Area under the cumulative distribution function (CDF) curve for k = 2–9. **(B)** CDF delta area for k = 2–9. **(C)** Tracking plot for k = 2–9. **(D)** Consensus clustering matrix for k = 2. **(E)** Kaplan–Meier survival analysis of two subgroups. **(F)** Heat-map of PRGs and the clinical characteristics between the two clusters.

To investigate whether differentially expressed PRGs had an impact on survival outcomes, we carried out the consensus clustering analysis of 249 KIRP patients. Based on the above PRGs, the results showed that the clustering variable (k) = 2 was considered to have the optimal stability from k = 2 to 9, implying that KIRP patients could be divided into two clusters (cluster 1 and cluster 2) with the highest intragroup correlations and the lowest intergroup correlations (Figures 2A–D). Notably, compared with those in cluster 1, KIRP patients in cluster 2 had a significantly longer survival (Figure 2E, *p* = 0.0041), indicating a significant prognostic value of these PRGs. Moreover, clinical characteristics including gender, age, and tumor TNM stage were presented in two clusters without significant differences (Figure 2F).

### Construction of a Prognostic Six-Gene Signature in KIRP Patients

**FIGURE 3:**
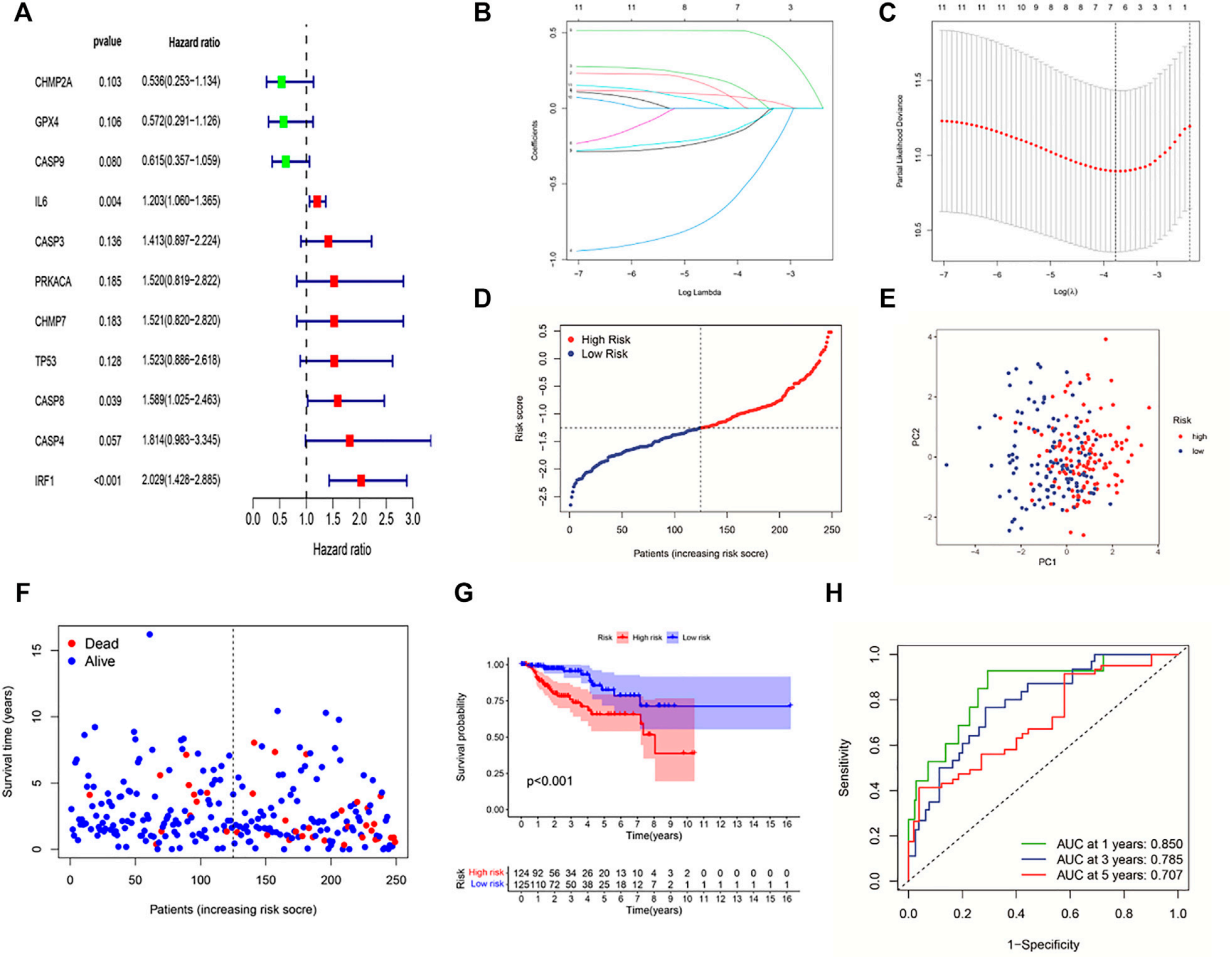
Construction of a six-gene prognostic signature in KIRP patients. **(A)** Univariate cox regression analysis of 11 PRGs (*p* < 0.2). **(B,C)** LASSO regression of 11 PRGs and the tuning parameter (λ) selection cross-validation curve. **(D)** Distribution of risk scores for KIRP patients. **(E)** PCA plot based on the risk score. **(F)** Distribution of patient survival status according to the high-risk group and the low-risk group. **(G)** Kaplan–Meier curves for OS in the low- and high-risk groups. **(H)** ROC curves to evaluate the predictive efficiency of the risk model.

The clinical implication of PRGs was further assessed in KIRP patients. As shown in univariate Cox regression analysis (Figure 3A), 11 (*IL6*, *CHMP2A*, *GPX4*, *CASP3*, *CASP4*, *CASP8*, *CASP9*, *CHMP7*, *PRKACA*, *TP53*, and *IRF1*) of PRGs were survival-related with *p* < 0.2. And then, LASSO-Cox regression analysis was performed using 11 prognostic genes, and a signature consisting of *CASP9*, *CHMP2A*, *GPX4*, *IL6*, *IRF1*, and *CASP8* was constructed based on the optimal λ score (Figures 3B,C). The risk score was calculated by the following formula: Risk score = (0.067*IL6 exp.) + (0.01011*CASP8 exp.) + (0.5066*IRF1 exp.) + (−0.4791*CASP9 exp.) + (−0.0988*CHMP2A exp.) + (−0.119*GPX4 exp.). 249 KIRP patients were approximately divided into the low-risk group and the high-risk group according to the median risk score (Figure 3D). As shown in Figure 3E, the result of principal component analysis (PCA) indicated patients of two risk groups could be distributed into two directions. Figure 3F shows that patients of the low-risk group tended to have a low probability of mortality compared to those of the high-risk group. Consistently, Kaplan–Meier analysis showed that the high-risk group had a significantly shorter survival time (Figure 3G, *p* < 0.001). A time-dependent ROC curve was performed to evaluate the predictive performance. And the AUC was 0.85 at 1 year, 0.785 at 2 years, and 0.707 at 3 years (Figure 3H), showing that this risk model exhibited high accuracy and sensitivity in predicting the prognosis of KIRP patients.

### Independent Prognostic Value of the Signature

**FIGURE 4:**
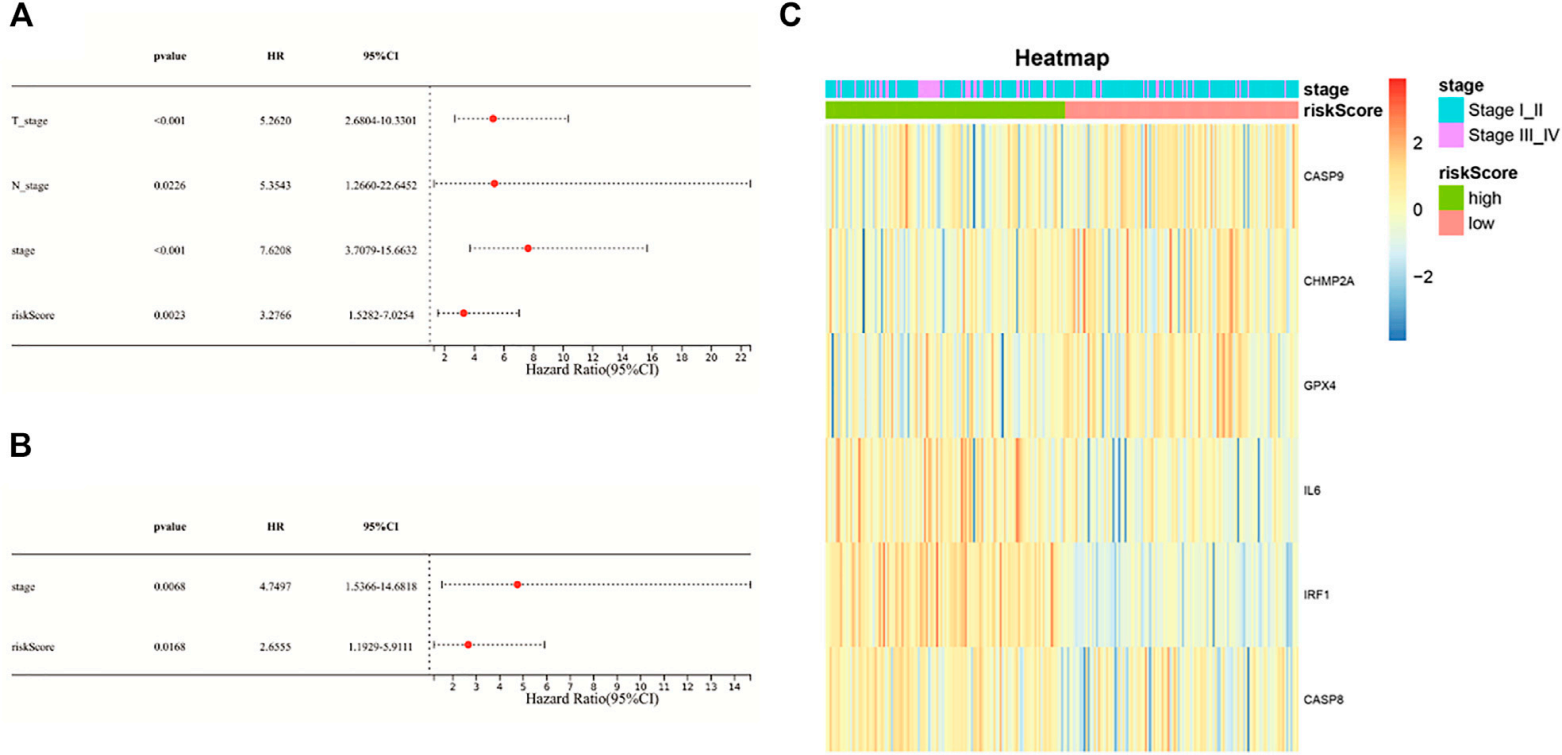
**(A)** Univariate Cox regression analysis of the risk score and other clinical characteristics associated with overall survival; **(B)** multivariate Cox regression analysis of the risk score and other clinical characteristics; **(C)** heat-map showing the relationship of the risk groups and tumor stage.

Univariate Cox regression analysis and multivariable Cox regression analysis were carried out to determine whether the risk score could serve as the independent prognostic predictor for survival in KIRP. The univariate Cox regression analysis showed that the risk score was significantly associated with poor survival (HR: 3.276, 95% CI 1.528–7.025, *p* = 0.002, Figure 4A). Moreover, other clinical characteristics including tumor stage and N-stage were found as the risk factors as well. After the adjustment of confounding factors, the result of multivariable Cox regression analysis suggested that the risk score was still a risk prognostic factor (HR: 2.655, 95% CI 1.192–5.911, *p* = 0.016, Figure 4B). As shown in Figure 4C, the patients suffering from advanced tumor stage had a higher probability of high risk score.

### Functional Enrichment Based on the Signature

**FIGURE 5:**
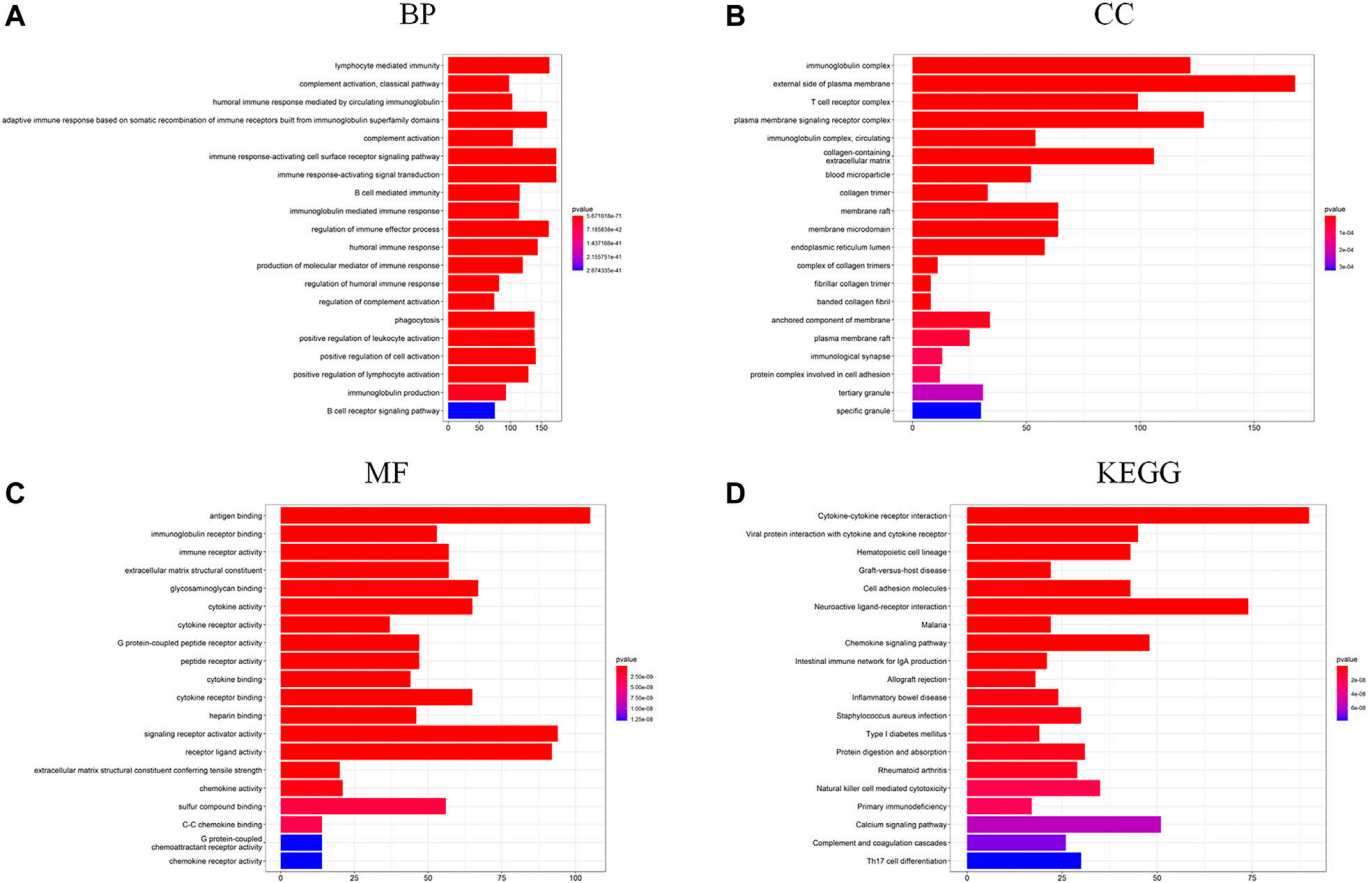
**(A–C)** GO functional enrichment analysis of DEGs in the two risk groups (BP, biological process; CC, cellular component; MF, molecular function); **(D)** KEGG pathway analysis of DEGs in the two risk groups.

To further investigate the differences in biological function and pathway between the low-risk group and the high-risk group, DEGs were generated using the “limma” R package. Then, these DEGs were further analyzed with the GO term and KEGG pathway enrichment analysis. As presented in Figure 5A, the top-rank biological processes were lymphocyte mediated immunity, complement activation pathway, and humoral immune response mediated by circulating immunoglobulin. Moreover, the most highly enriched cellular components associated with DEGs were immunoglobulin complex, external side of plasma membrane, and T cell receptor complex (Figure 5B). As for the molecular functions, antigen binding, immunoglobulin receptor binding, and immune receptor activity were on the top list (Figure 5C). Furthermore, the KEGG pathway analysis is shown in Figure 5D, and the cytokine-cytokine receptor interaction pathway and the viral protein interaction with cytokine and cytokine receptor signaling pathway were mostly associated with these DEGs. These results showed that these DEGs were significantly enriched in immune-related functions or pathways.

### Immune Characteristic Analysis Based on Pyroptosis-Related Risk Score

**FIGURE 6:**
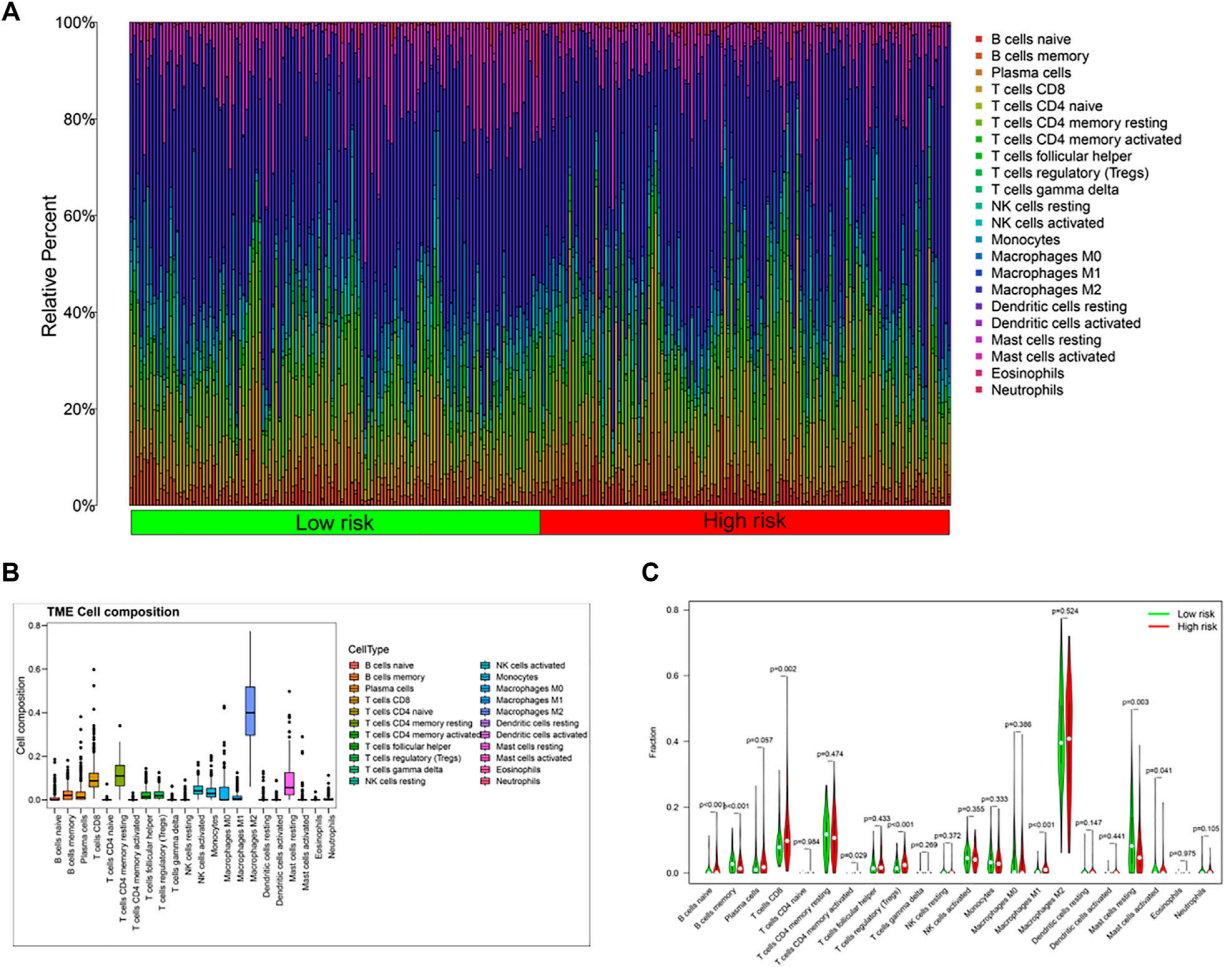
**(A,C)** Differences in immune cell composition between the high-risk group and the low-risk group; **(B)** tumor microenvironment immune cell composition in KIRP patients.

Based on functional enrichment, we speculated that tumor immune status of KIRP played an important role in the development of cancer. Therefore, the tumor immune microenvironment (TIM) of KIRP was further explored. Firstly, the abundance of immune cell infiltration was investigated. The overview of tumor microenvironment immune cell compositions is shown in Figure 6B, in which 22 type cells had differential distributions in KIRP. To be specific, M2 macrophages were found with an especially high infiltration level. The infiltration levels of naïve B cells, CD8^+^ T cells, regulatory T cells, and M1 macrophages in the high-risk group were significantly upregulated, while the infiltration levels of memory B cells, activated mast cells, and resting mast cells decreased (Figures 6A,C, *p* < 0.05).

### Identification and Validation of the Hub Genes *In Vitro*

**FIGURE 7:**
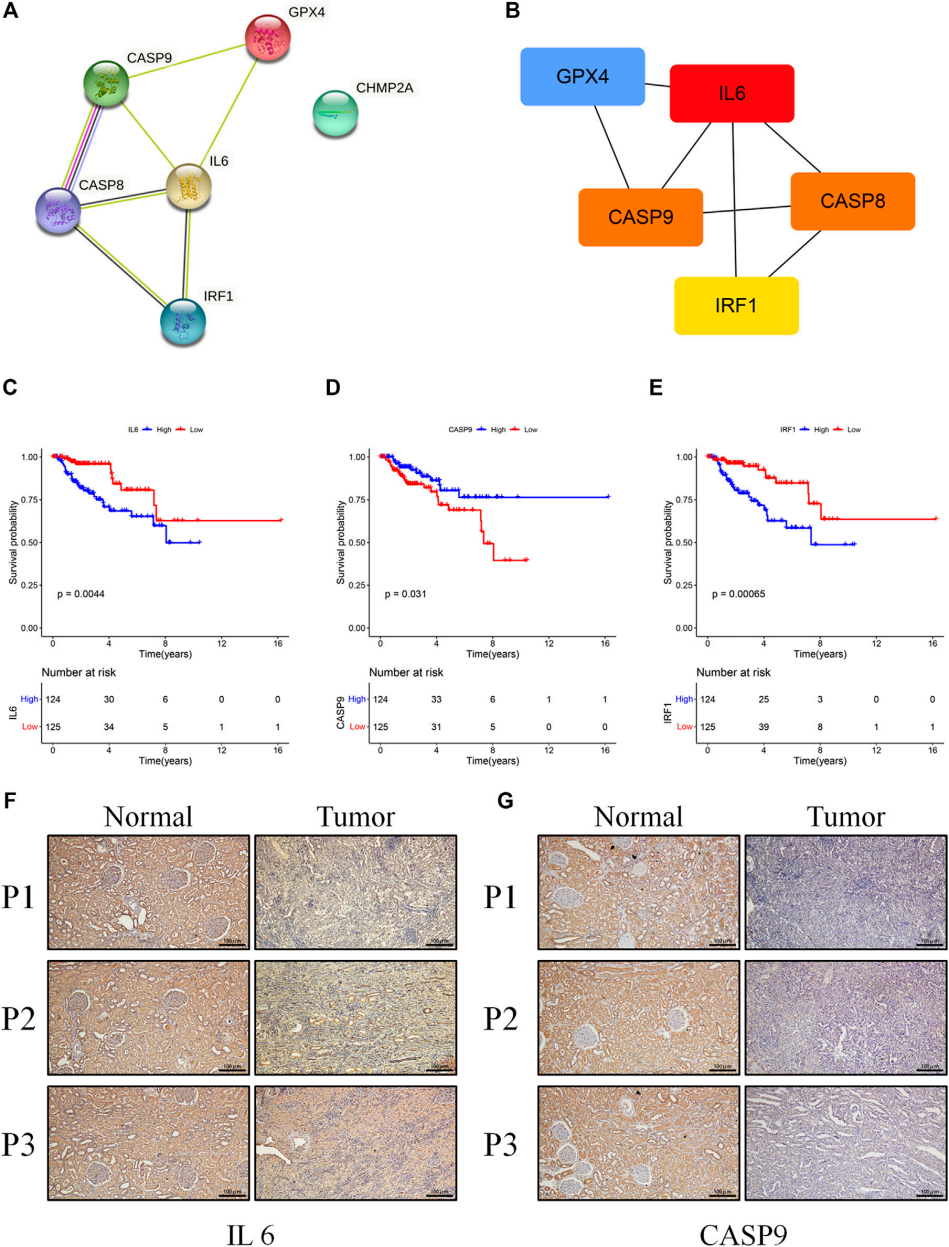
**(A)** The PPI network was constructed containing six genes of the signature. **(B)** Screening hub genes from the PPI network (red node: genes with a high MCC score; blue node: genes with a low MCC score). **(C–E)** The cohort was divided into two groups (high and low) according to their median expression value separately, and the expressions of *IL6*, *RAF1*, and *CASP9* were associated with overall survival (*p* < 0.05). **(F,G)** Results of *IL6* and *CASP9* in immuno-histochemical staining between KIRP and normal tissues (scale bar values: 100 µm).

To further explore genetic interrelationships in the PRG signature, the PPI network of these genes was obtained using the STRING database (Figure 7A). *IL6* was the hub node in the obtained interactive network. Next, the CytoHubba plugin in Cytoscape was used, and the genes with the top three MCC values (*IL6*, *CASP8*, and *CASP9*) were identified as candidate hub genes (Figure 7B). Meanwhile, we assessed the prognostic role of these PRGs in the signature, and three genes (*CASP9*, *IL6*, and *IRF1*) were survival-related (*p* < 0.05). High expression of *CASP9* was significantly correlated with longer overall survival in KIRP patients (*p* = 0.031), while high *IL6* (*p* = 0.004) and *IRF1* (*p* < 0.001) expressions had a shorter survival time (Figures 7C–E). The intersections of the above genes were selected as hub genes. Finally, two hub genes (*IL6* and *CASP9*) were identified and further validated through protein expression levels *in vitro*. As depicted in immuno-histochemical staining, the protein expression levels of *IL6* and *CASP9* were significantly downregulated in KIRP tissues compared with normal renal tissues (Figures 7F,G).

## Discussion

Pyroptosis has been defined as an inflammasome-induced programmed cell death, and it was initially observed in immune defense and anti-infection for eliminating viral and bacterial infections (Zychlinsky et al., 1992). Recently, increasing studies have demonstrated that pyroptosis played a vital role in carcinogenesis, and inducing tumor cell pyroptosis might be a potential treatment strategy for cancers (Wang et al., 2019). However, the role of pyroptosis in KIRP patients remains unclear.

In the present study, we comprehensively evaluated the mRNA expression levels of 52 PRGs in KIRP and normal tissues, from which 20 genes were differentially expressed. These patients were categorized into two groups based on consensus clustering analysis. Patients in cluster 2 had a longer survival time than those in cluster 1, implying that these PRGs might be important for predicting the prognosis of KIRP patients. Subsequently, using univariate Cox regression analysis and LASSO-Cox regression analysis, we had constructed a six-gene risk model with good prediction performance in the survival of KIRP. The results showed that patients in the high-risk group had a poor survival outcome and the risk score was an independent prognostic factor. Functional analysis using GO/KEGG analysis indicated that DEGs between the high-risk group and the low-risk group were closely associated with immune functions or pathways. Following that, we further explored the TIM of KIRP, showing a high infiltration level of M2 macrophages and differential distributions of immune cells between two risk groups. Finally, we identified two hub genes (*IL6* and *CASP9*), which were validated *via* protein expression levels *in vitro*.

For genes within the constructed signature, caspase-9 encoded by *CASP9* is a caspase trigger point, which plays an important role in the GSDME-mediated pyroptosis pathway. Caspase-9 activation can trigger caspase-3, inducing GSDME-mediated pyroptosis. Recent studies demonstrated that caspase-9 could be activated by the Tom20/Bax/Cytochrome c pathway in melanoma or by lobaplatin/ROS and JNK phosphorylation/Bax/Cytochrome c pathways in colon cancer, showing a great potential value of clinical application (Zhou et al., 2018; Yu et al., 2019). In this study, we found that the mRNA expression of *CASP9* was significantly decreased in KIRP tissues, and patients with high *CASP9* expression levels were correlated with longer overall survival. Further research studies observed that the methylation level of *CASP9* promoter was significantly elevated in KIRP (Supplementary Figure S1, *p* < 0.001) and thus reduced the expression of the gene. Therefore, *CASP9* was a protective gene and might be a potential therapeutic target for KIRP. *IL6* is a cytokine involved in numerous biological processes including immune response, inflammation, and embryonic development, and it is also a key factor in tumor development and progression (Hirano, 2021). For example, *IL6* could promote the development and proliferation of pancreatic cancer cells through the STAT3–Pim kinase axis (Block et al., 2012). Lippitz et al. reported that serum *IL6* was positively correlated with tumor stage or metastases, and increased *IL6* meant poor survival outcomes (Lippitz and Harris, 2016). We also obtained a similar result (*p* = 0.031), showing that it was a prognostic risk factor. Notably, the mRNA expression of *IL6* was downregulated in KIRP. Caspase8, encoded by *CASP8*, was proved to activate caspase-1 and *GSDMD* cleavage, thus resulting in pyroptosis (Orning et al., 2018; Han et al., 2021). Additionally, *IRF1* was considered a transcription factor regulating pyroptosis. Recent studies had proved that *IRF1* could transcriptionally induce *GSDMD* expression for pyroptotic cell death (Karki et al., 2020). In our study, *IRF1* was also downregulated in KIRP. Kang et al. demonstrated that conditional *GPX4* knockout could trigger lipid peroxidation–dependent caspase-11 and *GSDMD* cleavage, leading to pyroptosis (Kang et al., 2018). Additionally, SU et al. observed that high expression of *GPX4* in ccRCC promoted cancer cell proliferation and metastasis *in vitro* (Su et al., 2019). Here, *GPX4* was found with high expression in KIRP tissues and increased significantly in the high-risk group. Given the role of *GPX4*, it could also serve as a therapeutic target. However, the role of *CHMP2A* in pyroptosis is largely unclear and deserves further exploration.

According to DEGs between the high-risk group and the low-risk group, functional enrichment analysis in GO/KEGG showed that immune functions or immune-related pathways were highly frequent, such as activation of immune response and adaptive immune response, which meant that the TIM might be the key to the KIRP progression. Then, a high infiltration level of M2 macrophages was observed in the tumor microenvironment of KIRP, which was related to the immunosuppression state. Existing studies have demonstrated tumor-associated M2 macrophages could promote cell proliferation and angiogenesis and accelerate tumor progression (Fan et al., 2021; Xie et al., 2021). This might be a part of reasons that KIRP patients obtained poor therapeutic effect from immune checkpoint inhibitors. The activated M1 macrophages could produce inflammatory cytokines, for example, TNF-α, IL-1, and IL-12, enhance T cell function, and then exert antitumor functions (Yang et al., 2021). Moreover, activated T cells and B cells play protective roles in tumor immunity (Lin et al., 2013). Conversely, regulatory T cells and mast cells exert negative effects in antitumor (Maciel et al., 2015; Hirano, 2021). However, compared with the low-risk group, the tumor-protective immune cells, such as M1 macrophages and CD8 + T cells, were increased in the high-risk group. The TIM is a complex and disordered process in the development of tumor, which needs further research.

To our knowledge, this is the first time to systemically explore the relationship of PRGs and KIRP. The above results might provide novel insights into predicting prognostic, clinical decision-making and future research studies of KIRP. However, some limitations exist in this study. Firstly, the pyroptosis-related risk model in KIRP was constructed based on TCGA database. Due to the lack of appropriate datasets, this risk prognostic model could not be verified by other databases. However, its prognostic value and robustness were proved *via* different methods. Secondly, in the process of constructing this model, we might have excluded other prognostic genes which were not associated with pyroptosis. Finally, although immuno-histochemical staining was performed to validate some differentially expressed PRGs, more fundamental experiments to elucidate the role of these genes were encouraged.

## Conclusion

In conclusion, a prognostic model based on six PRGs was constructed, which could serve as an independent prognostic factor for KIRP patients. And the level of tumor immune cell infiltration was significantly different between the low-risk group and the high-risk group. Finally, two hub genes were identified and validated *in vitro*. These primary results might provide some useful value for the clinical prognosis and future research studies of KIRP.

## Data Availability

The datasets presented in this study can be found in online repositories. The names of the repository/repositories and accession number(s) can be found in the article/Supplementary Material.
