# Supplementary material for: A Novel Pyroptosis-Related Gene Signature for Predicting Prognosis in Kidney Renal Papillary Cell Carcinoma
Source: Front Genet. 2022 Mar 23;13:851384. doi: 10.3389/fgene.2022.851384 (PMC8984942; doi:10.3389/fgene.2022.851384)

**Supplementary Figure S1.** The methylation level of *CASP9* promoter between KIRP and normal tissues (P = 0.0001) from the UALCAN database (http://ualcan.path.uab.edu/).


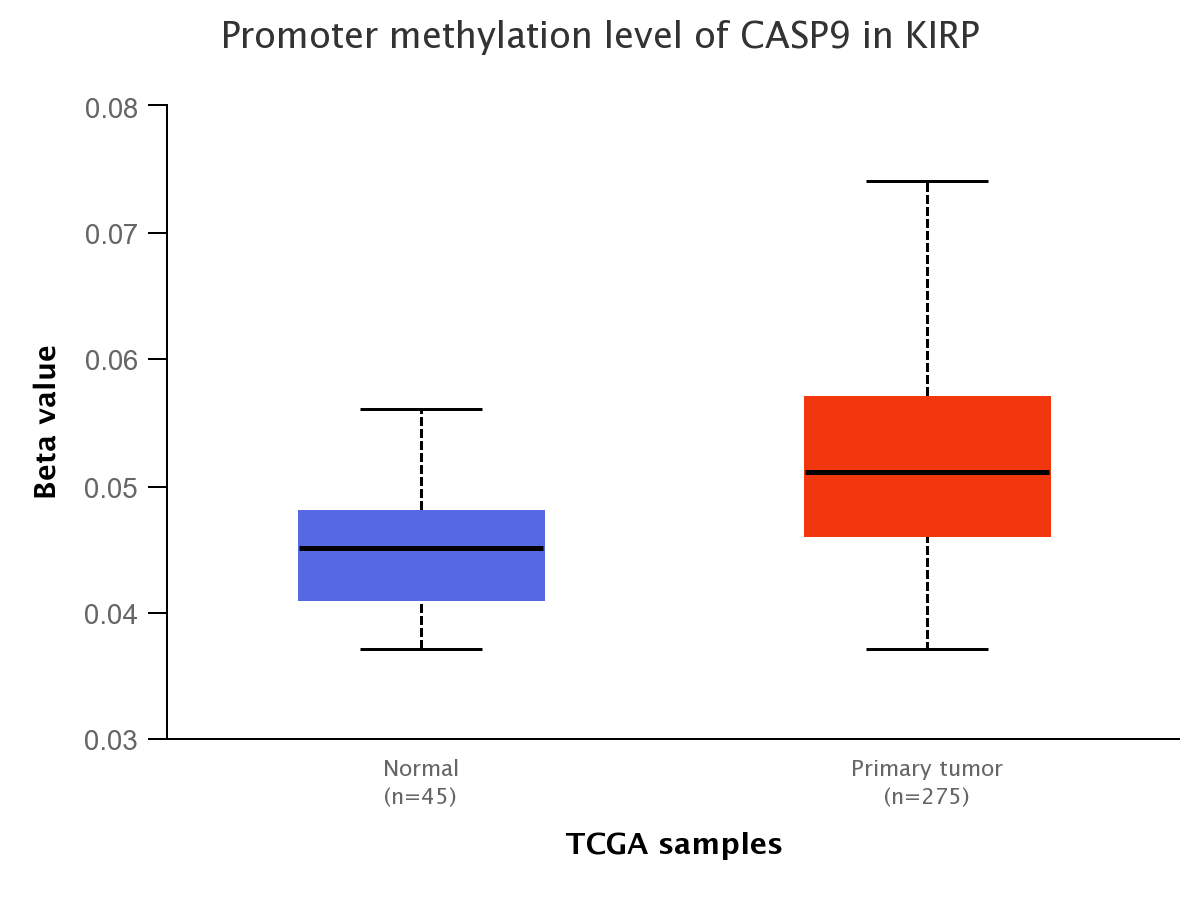

Supplement: Supplementary file 1 [file Table1.DOCX]
