# Supplementary material for: A Novel Pyroptosis-Related Gene Signature for Predicting Prognosis in Kidney Renal Papillary Cell Carcinoma
Source: Front Genet. 2022 Mar 23;13:851384. doi: 10.3389/fgene.2022.851384 (PMC8984942; doi:10.3389/fgene.2022.851384)
Supplement: Supplementary file 2 [file Table2.DOCX]

**Supplementary Table 1.** Summary of 52 pyroptosis-related genes in KIRP patients.

| Genes | Genes | Genes | Genes | Genes | Genes |
| --- | --- | --- | --- | --- | --- |
| *AIM2* | *CASP8* | *CHMP6* | *GSDMD* | *IL6* | *NLRP7* |
| *BAK1* | *CASP9* | *CHMP7* | *GSDME* | *IRF1* | *NOD1* |
| *BAX* | *CHMP2A* | *CYCS* | *GZMA* | *IRF2* | *NOD2* |
| *CASP1* | *CHMP2B* | *ELANE* | *GZMB* | *NLRC4* | *PJVK* |
| *CASP3* | *CHMP3* | *GPX4* | *HMGB1* | *NLRP1* | *PLCG1* |
| *CASP4* | *CHMP4A* | *GSDMA* | *IL18* | *NLRP2* | *PRKACA* |
| *CASP5* | *CHMP4B* | *GSDMB* | *IL1A* | *NLRP3* | *PYCARD* |
| *CASP6* | *CHMP4C* | *GSDMC* | *IL1B* | *NLRP6* | *SCAF11* |
| *TIRAP* | *TNF* | *TP53* | *TP63* |  |  |
